# Supplementary material for: Examining the Change of Human Mobility Adherent to Social Restriction Policies and Its Effect on COVID-19 Cases in Australia
Source: Int J Environ Res Public Health. 2020 Oct 29;17(21):7930. doi: 10.3390/ijerph17217930 (PMC7662641; doi:10.3390/ijerph17217930)
Supplement: Supplementary file 1 [file ijerph-17-07930-s001.pdf]

Supplementary Materials: The following are available online at www.mdpi.com/xxx/s1.

Table S1. Correlation coefficients

	1st Wave									2nd Wave
	AUS	NSW	VIC	QLD	WA	SA	NT	TAS	ACT	VIC
Between growth rate and mobility change										
Lockdown	**0.494**	**0.406**	**0.551**	**0.435**	**0.420**	**0.411**	-0.039	**0.337**	**0.508**	-0.241
7 Days after lockdown	**0.780**	**0.733**	**0.812**	**0.615**	**0.627**	**0.601**	0.176	**0.553**	**0.573**	-0.233
14 Days after lockdown	**0.792**	**0.777**	**0.812**	**0.625**	**0.598**	**0.633**	**0.233**	**0.531**	**0.478**	-0.085
Between doubling time and mobility change										
Lockdown	0.162	0.038	-0.121	0.105	-0.115	-0.190	**-0.314**	**-0.272**	-0.191	**0.369**
7 Days after lockdown	-0.041	-0.155	**-0.293**	-0.057	**-0.244**	**-0.303**	-0.135	**-0.276**	-0.042	**0.326**
14 Days after lockdown	**-0.242**	**-0.235**	**-0.339**	-0.189	**-0.225**	-0.138	0.128	**-0.286**	-0.028	0.167

Note: values in bold indicate $p<0.01$.

Table S2. R square of each regression model

	1st Wave									2nd Wave
	AUS	NSW	VIC	QLD	WA	SA	NT	TAS	ACT	VIC
Growth rate as the dependent variable										
Lockdown	0.578	0.481	0.560	0.656	0.581	0.476	0.040	0.309	0.493	0.525
7 Days after lockdown	0.867	0.769	0.907	0.881	0.807	0.814	0.339	0.68	0.792	0.262
14 Days after lockdown	0.923	0.880	0.881	0.704	0.610	0.649	0.162	0.489	0.496	0.136
Doubling time as the dependent variable										
Lockdown	0.302	0.042	0.088	0.145	0.082	0.250	0.084	0.207	0.097	0.412
7 Days after lockdown	0.237	0.067	0.398	0.272	0.391	0.429	0.278	0.441	0.373	0.253
14 Days after lockdown	0.214	0.094	0.159	0.101	0.086	0.138	0.116	0.223	0.116	0.182

Table S3-1. Coefficients of each type of mobility with growth rate as the dependent variable

		1st Wave									2nd Wave
		AUS	NSW	VIC	QLD	WA	SA	NT	TAS	ACT	VIC
Lockdown	RetailRecreation	**-1.227**	**-0.841**	**-0.966**	**-1.037**	**-0.725**	**-1.186**	-0.108	-0.401	**-0.609**	1.115
	GroceryPharmacy	**0.761**	**0.864**	**0.513**	**0.558**	0.172	**0.675**	0.140	-0.298	**0.679**	0.391
	Parks	0.075	0.029	0.107	-0.139	-0.129	0.096	-0.232	0.171	-0.026	0.134
	TransitStations	**1.141**	0.642	**1.159**	**1.402**	**1.434**	**0.840**	0.240	**0.879**	0.614	**-2.352**
	Workplaces	**0.036**	0.130	**0.074**	**-0.334**	-0.269	-0.017	-0.249	-0.060	-0.140	**1.019**
	Residence	0.213	0.315	0.237	-0.068	0.160	-0.133	-0.060	-0.060	-0.164	0.335
7 Days after lockdown	RetailRecreation	-0.360	-0.339	-0.200	-0.101	-0.159	**-0.700**	0.372	-0.051	-0.394	1.304
	GroceryPharmacy	**0.325**	**0.375**	0.177	0.050	0.058	**0.529**	-0.002	-0.022	**0.354**	-0.042
	Parks	**-0.259**	**-0.231**	-0.108	**-0.186**	**-0.320**	-0.031	-0.361	-0.425	-0.129	-0.214
	TransitStations	**1.862**	**1.724**	**1.522**	**1.402**	**1.526**	**1.434**	0.366	**1.439**	**1.585**	**-1.563**
	Workplaces	**-0.737**	-0.455	**-0.482**	**-0.623**	**-0.609**	**-0.843**	-0.480	-0.369	**-1.181**	0.656
	Residence	0.047	0.346	0.129	-0.112	0.008	-0.226	-0.094	0.063	-0.296	0.193
14	RetailRecreation	**0.524**	0.093	0.407	0.330	0.355	0.419	0.417	0.280	-0.104	1.285

		AUS	NSW	VIC	QLD	WA	SA	NT	TAS	ACT	VIC
	GroceryPharmacy	**-0.369**	**-0.252**	**-0.286**	-0.286	-0.244	-0.276	-0.286	-0.417	-0.116	-0.245
	Parks	**-0.252**	-0.124	**-0.191**	-0.154	-0.152	0.072	-0.006	0.390	-0.187	-0.166
	TransitStations	**1.595**	**1.672**	**1.431**	**1.132**	**1.271**	**0.748**	0.308	0.201	**1.330**	-1.095
	Workplaces	**-0.633**	-0.300	**-0.660**	-0.519	**-0.769**	**-0.534**	-0.162	-0.526	**-1.055**	0.627
	Residence	0.145	0.348	-0.022	-0.081	0.023	-0.139	0.079	-0.580	-0.478	0.232

Note: values in bold indicate $p<0.01$.

Table S3-2. Coefficients of each type of mobility with doubling time as the dependent variable

		1st Wave									2nd Wave
		AUS	NSW	VIC	QLD	WA	SA	NT	TAS	ACT	VIC
Lockdown	RetailRecreation	**1.227**	0.510	0.447	0.671	0.092	0.028	-0.491	**-1.226**	-0.123	-0.588
	GroceryPharmacy	**-0.842**	-0.100	-0.131	-0.315	-0.212	**0.629**	0.344	**0.650**	0.273	-0.275
	Parks	0.005	-0.056	-0.085	0.189	0.013	0.113	-0.164	0.098	-0.016	-0.253
	TransitStations	-0.502	-0.348	-0.435	-0.351	-0.032	-0.294	0.005	-0.061	-0.087	**1.853**
	Workplaces	0.066	0.065	-0.365	-0.263	-0.494	-0.340	-0.242	-0.285	-0.240	**-0.829**
	Residence	-0.177	0.056	-0.388	-0.192	-0.483	0.299	-0.277	-0.569	-0.007	-0.276
7 Days after lockdown	RetailRecreation	0.745	0.572	-0.309	0.309	0.492	-0.134	-0.417	-0.766	-0.293	-1.177
	GroceryPharmacy	**-0.574**	-0.176	-0.114	-0.161	-0.182	0.104	0.148	0.378	0.309	0.26
	Parks	0.441	-0.223	0.258	0.125	**-0.427**	0.005	-0.293	0.090	**0.331**	0.141
	TransitStations	**-1.432**	-0.541	-0.007	-0.547	-0.078	0.304	0.173	0.458	0.089	**1.244**
	Workplaces	**1.056**	-0.245	-0.075	0.049	-0.123	0.111	-0.519	-0.037	0.230	-0.886
	Residence	0.243	-0.354	0.016	-0.162	0.143	0.709	-0.604	0.400	0.524	-0.46
14 Days after lockdown	RetailRecreation	**1.314**	0.435	0.622	0.444	0.127	-0.570	0.195	-0.315	-0.980	-1.137
	GroceryPharmacy	-0.423	-0.070	-0.167	-0.253	0.013	0.272	0.113	0.123	**0.540**	0.459
	Parks	0.003	0.046	-0.051	0.090	0.116	0.276	-0.410	-0.262	-0.097	-0.094
	TransitStations	**-1.647**	-0.409	-0.929	-0.753	-0.571	0.116	0.382	**1.165**	0.619	0.925
	Workplaces	-0.037	-0.440	0.048	0.419	0.321	0.689	**-0.735**	-0.102	-0.124	**-1.097**
	Residence	-0.465	-0.186	-0.088	0.190	0.167	0.738	-0.421	0.932	-0.098	-0.559

Note: values in bold indicate $p<0.01$.
